# Gap between pediatric and adult approvals of molecular targeted drugs

**DOI:** 10.1038/s41598-020-73028-w

**Published:** 2020-10-13

**Authors:** Satoshi Nishiwaki, Yuichi Ando

**Affiliations:** Department of Advanced Medicine, Nagoya University Hospital, 65 Tsurumai-cho, Showa-ku, Nagoya, 466-8560 Japan

**Keywords:** Drug regulation, Drug development

## Abstract

To clarify the approval status of molecular targeted antineoplastic drugs in the United States (U.S.), the European Union (E.U.), and Japan (JP), we checked the status of pediatric indications according to the package insert of each drug. A total of 103 drugs were approved for adult patients in at least one of the three regions whereas only 19 drugs were approved for pediatric patients. Sixty-six of 103 drugs (64.1%) had adult indications in the U.S., the E.U., and JP, whereas only three drugs had pediatric indications in all three regions. Abnormalities in six genes (NRAS, ABL1, JAK2, KIT, ALK and BRAF) were common in childhood cancers as well as adult cancers, for which at least one approved drug could be a potentially actionable drug. Although there were 16 candidate drugs that had adult indications for these abnormalities, only three drugs (18.8%) had pediatric indications. We confirmed that there were few molecular targeted antineoplastic drugs with pediatric indications in the U.S., the E.U., and JP compared with the number of approved drugs for adults. Drugs targeting genomic abnormalities which were common in both adult and pediatric cancers were considered to be good candidates for expansion of their indication for pediatric patients.

## Introduction

Recent technological advances in genomics made it possible to rapidly identify the genetic abnormalities underlying a single patient’s cancer, which led to precision medicine^1^. Genomic abnormalities can be tested in pediatric cancer patients, as well as in adult cancer patients. The identification of genomic abnormalities can lead to the development of molecular targeted therapies. Although efforts have been made in the pediatric oncology community^2–4^, there remain few molecular targeted drugs with pediatric indications.

Large-scale studies have shown the landscape of genomic abnormalities in childhood cancers^5,6^. Although the frequency and type of genomic abnormalities differs somewhat between adults and children, there are some genomic abnormalities that are relatively common in both adults and children. It is important to take both the frequency of genomic abnormalities and the availability of potentially actionable approved drugs into consideration. The purpose of this study was to confirm the current status of pediatric indications for molecular targeted drugs and to consider strategies that could lead to more efficient pediatric drug development based on the frequency of genomic abnormalities in childhood cancers.

## Methods

### Data sources

We listed molecular targeted antineoplastic drugs using data from the Kyoto Encyclopedia of Genes and Genomes (KEGG)^7^. The status of pediatric and adult indications was confirmed by checking prescribing information from the U.S. Food and Drug Administration (FDA) (https://www.accessdata.fda.gov/scripts/cder/daf/), summaries of product characteristics from the European Medicines Agency (EMA) (https://www.ema.europa.eu/en), and package inserts of the Pharmaceuticals and Medical Devices Agency (PMDA) (https://www.pmda.go.jp/PmdaSearch/iyakuSearch/) as of February 2020.

We selected candidate genes according to two previous large studies into childhood cancers^5,6^. To assess potentially actionable drugs, we used evidence based on OncoKB (https://oncokb.org)^8^, CanDL (https://candl.osu.edu/browse)^9^, and Japanese clinical practice guidance (J-ClinG)^10^.

### Definition

We accessed “New Drug Approvals in the USA, Europe and Japan (https://www.genome.jp/kegg/drug/br08328.html)” and “Antineoplastics (https://www.genome.jp/kegg/drug/br08340.html)” in KEGG, and selected molecularly targeted agents. The anatomical therapeutic chemical (ATC) codes were L01XC “Monoclonal antibodies” [including alemtuzumab (L04AA34) and ibritumomab (V10XX02)], L01XE “Protein kinase inhibitors”, or L01XX “Other antineoplastic agents”. A pediatric indication was determined if there was an indication for at least one neoplasm under the age of 15 years. It was judged that a drug had no pediatric indication if there was “no description of indications and dosage for children” and “a statement that safety and efficacy in pediatric patients have not been established” in prescribing information from the FDA (1 INDICATIONS AND USAGE, 2 DOSAGE AND ADMINISTRATION and 8 USE IN SPECIFIC POPULATIONS-8.4 Pediatric Use), summaries of product characteristics from the EMA (4. CLINICAL PARTICULARS-4.1 Therapeutic indications and 4.2 Posology and method of administration), or package inserts with instructions from the PMDA (dosage and administration; administration to children, etc.).

Based on the idea to efficiently expand pediatric indication among molecular targeted drugs approved for adults, we selected candidate genes according to two previous large studies into childhood cancers^5,6^: 25 genes which were commonly mutated across age groups^5^ and 51 genes from a pediatric pan-cancer study^6^. Fifteen genes overlapped, therefore the level of evidence was examined for 61 genes (supplemental Table S1). The incidence of the 51 genes from the pediatric pan-cancer study^6^ was normalized according to the incidence of childhood cancer^11^. Potentially actionable drugs for a specific gene, where genetic lesions were actionable, were considered to be good candidates when there was at least clinical evidence for the drug in all three references: Level 3 or higher in OncoKB, Tier 2 or higher in CanDL and Therapeutic Efficacy 3A or higher in J-ClinG. The type of cancer was not considered.

**Table 1:** List of molecular targeted antineoplastic drugs with pediatric indications.

ATC	Active ingredient	Pediatric indication		
		U.S	E.U	JP
L01XC02	Rituximab	N	N	Y
L01XC05	Gemtuzumab ozogamicin	Y	N	N
L01XC11	Ipilimumab	Y	Y	N
L01XC12	Brentuximab vedotin	N	N	Y
L01XC16	Dinutuximab	Y	Y	NA
L01XC17	Nivolumab	Y	N	N
L01XC18	Pembrolizumab	Y	N	N
L01XC19	Blinatumomab	Y	Y	Y
L01XC31	Avelumab	Y	N	N
L01XE01	Imatinib mesylate	Y	Y	N
L01XE06	Dasatinib	Y	Y	N
L01XE08	Nilotinib hydrochloride hydrate	Y	Y	Y
L01XE10	Everolimus	Y	Y	Y
L01XE12	Vandetanib	N	Y	N
L01XE53	Larotrectinib sulfate	Y	Y	NA
L01XE56	Entrectinib	Y	NA	Y
L01XX14	Tretinoin	Y	NA	Y
L01XX27	Arsenic trioxide	Y	N	Y
L01XX67	Tagraxofusp	Y	NA	NA

*ATC* The anatomical therapeutic chemical codes; *U.S.* United States; *E.U.* European Union; *JP* Japan; *Y* Yes; *N* No; *NA* Not approved for either adults or children (as of February 2020).

## Results

### Pediatric indications in the U.S., the E.U., or JP

**Figure 1:**
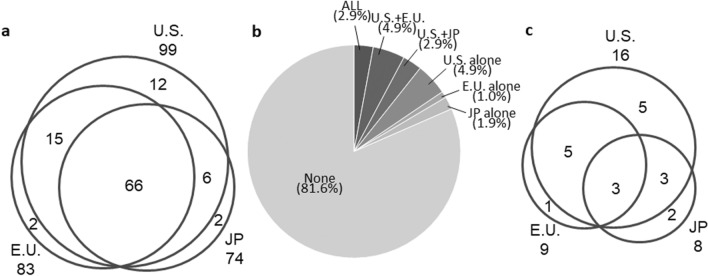
Approval status of molecular targeted antineoplastic drugs in the U.S., the E.U., or JP. **a** 103 drugs with adult indications. **b** Overview of pediatric indications. **c** 19 drugs with pediatric indications. *U.S.* United States; *E.U* European Union; *JP* Japan.

A total of 103 molecular targeted drugs were approved for adults in the U.S., the E.U., or JP. Sixty-six drugs (64.1%) were approved in all three regions whereas 16 drugs (15.5%) were only approved in one of the three regions (Fig. 1a).

Nineteen of 103 approved drugs (18.4%) had pediatric indications. Only three of 19 drugs (15.8%) (blinatumomab, nilotinib, and everolimus) had pediatric indications in all three regions whereas eight drugs (42.1%) only had pediatric indications in one of the three regions (Table 1, Fig. 1b,c).

### Candidates for expanding pediatric indications

Of 61 candidate genes, 24 genes (39.3%) had at least one approved drug which had clinical evidence in at least one of the three references, while six genes (NRAS, ABL1, JAK2, KIT, ALK and BRAF) (9.8%) had at least one approved drug that had clinical evidence in all three references (supplemental Table S1). Therefore, we considered potentially actionable drugs for these six genes to be good candidates. The total frequency of the six gene abnormalities was between 3.7% and 9.4%, according to the frequencies of each gene from past studies^5,6^. Potentially actionable drugs for each gene were summarized in Table 2. Although all drugs, except cobimetinib in JP, are approved for adults, only nilotinib had pediatric indications in all three regions, while dasatinib and imatinib had those in the U.S. and the E.U.

**Table 2:** Targeted genes and candidate drugs with high priority for development of pediatric indication.

Target gene	Frequency (%)*	Potentially actionable drug**	Approval status***		
			U.S	E.U	JP
NRAS	1–3.8	Binimetinib**^1^	A	A	A
ABL1	1.2	Dasatinib	A&P	A&P	A
		Imatinib	A&P	A&P	A
		Ponatinib	A	A	A
		Nilotinib	A&P	A&P	A&P
		Bostinib	A	A	A
JAK2	1	Ruxolitinib	A	A	A
KIT	0.5–1	Regorafenib	A	A	A
		Imatinib	A&P	A&P	A
		Sunitinib	A	A	A
ALK	0–1.9	Crizotinib	A	A	A
		Alectinib	A	A	A
		Ceritinib	A	A	A
BRAF	0–0.5	Dabrafenib + Trametinib	A	A	A
		Vemurafenib + Cobimetinib	A	A	NA
		Trametinib**^2^	A	A	A

*U.S.* United States. E.U. = European Union; *JP* Japan; *A* Adult indication; *A&P* Adult and pediatric indications; *NA* Not approved for either adults or children.

* Frequency of genomic abnormalities in childhood cancers.

** Not only for the primary target. Drugs with clinical evidence were listed according to the reference data base.

**^1^A MEK inhibitor which has clinical evidence for NRAS mutations.

**^2^A MEK inhibitor which has clinical evidence for BRAF alterations.

***Approval status as of February 2020.

## Discussion

This study revealed that there are only a few molecular targeted drugs with pediatric indications worldwide. A mechanism of action-based, biology-driven, pediatric oncology drug development was proposed to accelerate drug development and delivery of precision medicines to children^12^. Pediatric cancers have fewer mutations than adult cancers^5^, therefore, it is easier to identify their driver mutations. This suggests that appropriate molecular targeted drugs can be effective for pediatric patients, so it is desirable that molecular targeted drugs will receive proper approval for use in children. Although off-label use based on the best available evidence can realize fast access for individual children^13,14^, this should be avoided if possible because of the risk of adverse events^15^.

Legislation for pediatric drug development has been made in both the U.S. [Best Pharmaceuticals for Children Act (BPCA), Pediatric Research Equity Act (PREA)] and the E.U. [Regulation (EC) No 1901/2006], which requires pediatric studies to be undertaken, with some incentives for pharmaceutical companies. However, there are criteria that allow a waiver or a deferral in both the U.S. and the E.U. regulations^16,17^. These criteria include situations where the number of patients is low or the disease is unique to adults. Because the company was granted a class waiver, crizotinib was not developed for children under the EMA regulation^2,18^. The ALK inhibitors, crizotinib, alectinib, and ceritinib only have an indication for ALK-positive non-small cell lung cancer (NSCLC). It is known, however, that the incidence and organs affected differ between pediatric and adult cancers, and NSCLC is rare in children^11,19,20^. Although ALK was detected in 0% to 1.9% of childhood cancers, no ALK inhibitor has pediatric indications. ALK-positive pediatric anaplastic large cell lymphoma and inflammatory myofibroblastic tumor can be good candidates for ALK inhibitors^21^. In addition, the effect of ALK inhibitors could also be expected for central nervous system tumors such as neuroblastoma and glioblastoma considering efficacy of ALK inhibitors on NSCLC brain metastases^22^. Moreover, dabrafenib, a BRAF inhibitor which has adult indication for melanoma (all three regions) and NSCLC (the E.U. and JP) with a BRAF V600 mutation, showed a promising result in pediatric patients with BRAF V600 mutation-positive glioma in Phase I/IIa study^23^. In the era of cancer genomics, approval of anticancer drugs for each organ may be one of the obstacles to be overcome for pediatric indications. In 2017, Research to Accelerate Cures and Equity (RACE) for Children Act was enacted to require pediatric studies for new cancer drugs and the act could have increased pediatric study requirements from 0 to 78%^4^. The act goes into effect on August 18, 2020. After that, it requires pediatric evaluation of new molecular targeted drugs and biologics that are intended for the treatment of adult cancers and directed at a molecular target substantially relevant to the growth or progression of a pediatric cancer. In addition, it eliminates the PREA orphan exemption for pediatric studies for therapies that are directed at relevant molecular targets. It is necessary to pay close attention to whether this act will actually lead to expand indication in childhood cancers.

International harmonization and the use of new technologies is being explored for the development of pediatric drugs. The International Council for Harmonisation of Technical Requirements for Pharmaceuticals for Human Use (ICH) E11A guidelines for Pediatric Extrapolation is being discussed. “Modeling and Simulation” is one of the topics in the E11A guidelines. Recently, larotrectinib and entrectinib were granted pediatric indications, based on modeling and simulation data, for pediatric doses^24,25^. In addition, it should be noted that these drugs got a tumor-agnostic indication as long as solid tumors demonstrated neurotrophic tyrosine receptor kinase (NTRK) gene fusion in each region (larotrectinib: the U.S. and the E.U., entrectinib: the U.S. and JP). The frequency of NTRK fusion throughout all cancer types is not always high^26^. However, because the frequency of the NTRK fusion gene was relatively high in some childhood cancers, indications could be obtained for children as well as adults. Historically, pembrolizumab was the first approved tumor-agnostic drug, which was an anti-PD-1 monoclonal antibody approved for solid tumors with microsatellite instability-high (MSI-H) in the U.S. in 2017 (also approved for solid tumors with MSI-H in JP in 2018)^27^. Approval of new drugs based on similar procedures may increase in the future.

Interestingly, there were more molecular targeted drugs approved for children only in one of three regions than those approved for adults (42.1% vs. 15.5%). The ideas toward the approval for children may vary by region. Children with cancers should not be treated as little adults^28^. This study does not rule out the development of molecular targeted drugs from a child-specific perspective. There are unique difficulties with setting doses in pediatric patients to take into account the various developmental stages of neonates, infants, children and adolescents. Frequencies of targets may be one of the important factors for drug development considering that two of three drugs with pediatric indications in all the three regions (blinatumomab and nilotinib) were drugs for hematological malignancies which were the most common neoplasms, accounting for about half of childhood tumors. Therefore, we proposed the prioritization of target drugs according to the frequency of genomic abnormalities across childhood cancers. We considered genes in which abnormalities were common to both pediatric and adult patients as good candidates for drug development. Surprisingly, only six genes were found to have clinical evidence in all three reference sources. This may lead to increases in the number of drugs with pediatric indications by prioritizing and focusing on specific drugs.

In the era of genomic medicine, it is necessary more than ever to eliminate differences between pediatric and adult indications of molecular targeted drugs. The current situation needs rapid improvement, so that genomic abnormalities can be tested in childhood cancers, for which there is a lack of approved drugs. Concrete and realistic strategies will lead to the proper delivery of molecular targeted drugs to pediatric patients.

## Supplementary information

Supplementary information
