# Supplementary material for: Gap between pediatric and adult approvals of molecular targeted drugs
Source: Sci Rep. 2020 Oct 13;10:17145. doi: 10.1038/s41598-020-73028-w (PMC7555892; doi:10.1038/s41598-020-73028-w)
Supplement: Supplementary file 1 — Supplementary information [file 41598_2020_73028_MOESM1_ESM.docx]

**Gap between pediatric and adult approvals of molecular targeted drugs**

**Satoshi Nishiwaki, Yuichi Ando**

**Supplemental Table S1** List of candidate genes and clinical evidence level

| Targeted genes | Evidence level | | |
| --- | --- | --- | --- |
|  | OnkoKB (Level) | CanDL (Tier) | J-ClinG (Therapeutic Efficacy) |
| ABL1 | 1 | 1 | 1A |
| AKT1 | N | 3 | N |
| ALK | 1 | 1 | 1A |
| AMER1 | N | N | N |
| APC | N | N | N |
| ARID1A | N | N | 3B |
| ATM | 4 | N | 2B |
| ATRX | N | N | N |
| BRAF | 1 | 1 | 1A |
| BTG1 | N | N | N |
| CBL | N | N | N |
| CCND2 | N | N | 3A |
| CCND3 | N | 3 | N |
| CDK4 | 2A | 3 | 3B |
| CDK6 | N | N | 3B |
| CDKN1B | N | N | N |
| CDKN2A | 4 | N | 3A |
| CDKN2B | N | N | N |
| CRLF2 | N | N | N |
| CSF3R | N | 3 | N |
| CTNNB1 | N | N | 2B |
| EPOR | N | N | N |
| FBXW7 | N | N | 3B |
| FGFR1 | 3A | N | N |
| FLT3 | 1 | 3 | 1B |
| IDH1 | 1 | 3 | 3B |
| IL7R | N | 3 | N |
| JAK1 | N | 4 | 3A |
| JAK2 | 3A | 1 | 1B |
| JAK3 | N | 3 | 3B |
| KDM6A | 4 | N | 3B |
| KIT | 1 | 1 | 2A |
| KMT2C | N | N | N |
| KMT2D | N | N | N |
| KRAS | 3A | 4 | N |
| NF1 | 4 | N | N |
| NF2 | N | N | 2A |
| NOTCH1 | N | N | N |
| NPM1 | N | N | N |
| NRAS | 3A | 2 | 1B |
| PCBP1 | N | N | N |
| PDGFRA | 1 | 3 | 3B |
| PDGFRB | 1 | N | 1B |
| PIK3CA | 1 | 4 | N |
| PIK3CD | 1 | N | N |
| PIK3R1 | N | 4 | N |
| PTEN | N | N | 3A |
| PTPN11 | N | 4 | N |
| PTPN2 | N | N | N |
| RB1 | N | N | N |
| RIT1 | N | 3 | N |
| SETD2 | N | N | N |
| SF3B1 | N | N | N |
| SH2B3 | N | N | N |
| STAT5B | N | 4 | N |
| TERT | N | N | N |
| TP53 | N | N | N |
| TSC1 | 1 | N | 2A |
| TSC2 | 1 | N | 3A |
| WT1 | N | N | N |
| XPO1 | N | N | N |
| *N* No clinical evidence | | | |
| OncoKB (https://www.oncokb.org/, accessed February 2020) | | | |
| Level 1: FDA-recognized biomarker predictive of response to an FDA approved drug in this indication | | | |
| Level 2A: Standard care biomarker predictive of response to an FDA approved drug in this indication | | | |
| Level 2B: Standard care biomarker predictive of response to an FDA approved drug in another indication but not standard care for this indication | | | |
| Level 3A: Compelling clinical evidence supports the biomarker as being predictive of response to a drug in this indication, but neither biomarker nor drug is standard care | | | |
| Level 3B: Compelling clinical evidence supports the biomarker as being predictive of response to a drug in another indication, but neither biomarker nor drug is standard care | | | |
| Level 4: Compelling biologic evidence supports the biomarker as being predictive of response to a drug, but neither biomarker nor drug is standard care | | | |
| CanDL (https://candl.osu.edu/search/, accessed February 2020) | | | |
| Tier 1: Alteration has matching FDA approved or NCCN recommended therapy | | | |
| Tier 2: Alteration has matching therapy based on evidence from clinical trials, case reports, or exceptional responders. | | | |
| Tier 3: Alteration predicts for response or resistance to therapy based on evidence from pre-clinical data (in vitro or in vivo models) | | | |
| Tier 4: Alteration is a putative oncogenic driver based on functional activation of a pathway | | | |
| Japanese Clinical practice guidance (J-ClinG) | | | |
| 1A: Biomarker (gene mutation) that is approved by regulatory authority as a companion diagnostic for the said cancer type | | | |
| 1B: Biomarker (gene mutation) that is approved by FDA as a companion diagnostic (or complementary diagnostic) for the said cancer type | | | |
| Biomarker (gene abnormality), with which consistent results have been obtained to support clinical usefulness of an anticancer agent in a prospective clinical study with biomarker-based patient selection, or in meta-analysis data for the specific cancer | | | |
| 2A: Biomarker (gene abnormality), with which results have been obtained to support clinical usefulness of an anticancer agent in subgroup analysis of a prospective clinical study for the said cancer type | | | |
| 2B: Biomarker (gene abnormality) approved by regulatory authority for other cancer type(s), or with which results have been obtained to support clinical usefulness of an anticancer agent | | | |
| 3A: Biomarker (gene abnormality), for which a correlation with clinical usefulness of an anticancer agent has been reported in scientific knowledge-based case reports, etc. | | | |
| 3B: Biomarker (gene abnormality), with which a correlation with therapeutic efficacy of an anticancer agent has been reported by pharmacodynamic evaluation in vitro and in vivo | | | |
| 4: Gene abnormality that is known to be involved in cancer | | | |
